# Relationship Between Physicochemical Parameters and Risk of Analgesics and Antibiotics of Effluents From Three Selected Hospitals in Kumasi Metropolis, Ghana

**DOI:** 10.1155/tswj/2891425

**Published:** 2025-05-10

**Authors:** Emma Kofua Nsafoah, Cindy Yaa Gyeniaw, Alhassan Sulemana, Bernard Fei-Baffoe, David Azanu, Kodwo Miezah, Kofi Sekyere Boateng, Daniel Nimako Amprako, Jonathan Nartey Hogarh, Kwame Ohene Buabeng

**Affiliations:** ^1^Department of Environmental Science, Kwame Nkrumah University of Science and Technology, Kumasi, Ghana; ^2^Science Department, Akrokerri College of Education, Akrokerri, Ghana; ^3^Department of Public Health Education, Akenten Appiah-Menka University of Skills Training and Entrepreneurial Development, Mampong, Ghana; ^4^KNUST Central Laboratory, Kwame Nkrumah University of Science and Technology, Kumasi, Ghana; ^5^Department of Pharmacy Practice, Kwame Nkrumah University of Science and Technology, Kumasi, Ghana; ^6^Department of Pharmacy Practice, School of Pharmacy, University of Health and Allied Sciences, Ho, Ghana

**Keywords:** analgesics, antibiotics, environmental risk, hospital effluents, pharmaceuticals

## Abstract

This study is aimed at ascertaining the relationship between environmental risks of analgesics (acetaminophen, diclofenac, and ibuprofen), methylxanthine (caffeine), and antibiotics (amoxicillin, ciprofloxacin, and metronidazole) and physicochemical parameters (temperature, pH, electrical conductivity, salinity, total dissolved salts, and turbidity) of three replicates of untreated effluents from three hospitals (Kwame Nkrumah University of Science and Technology [KNUST] Hospital, Kumasi South Hospital [KSH], and Komfo Anokye Teaching Hospital [KATH]) within Kumasi, Ghana. The samples were filtered, extracted by solid phase, and analyzed by PerkinElmer Flexar HPLC. Samples showed ambient temperature, around neutral pH, and high electrical conductivity, salinity, total dissolved salts, and turbidity. Acetaminophen and diclofenac were detected at concentrations of 40.00–44.00 and 77.00–553.00 *μ*g/L, respectively, in KNUST samples. Acetaminophen (266.00-510.00 *μ*g/L), caffeine (60.00–85.00 *μ*g/L), diclofenac (55.00–380.00 *μ*g/L), ciprofloxacin (44.00–45.00 *μ*g/L), and metronidazole (18.00–42.00 *μ*g/L) were detected in KSH samples. In KATH samples, acetaminophen and ciprofloxacin were found at concentrations of 29.00–114.00 and 74.00–232.00 *μ*g/L, respectively. Ibuprofen and amoxicillin in samples were below detection levels. A Pearson correlation showed an inverse relationship between temperature, pH, and acetaminophen; a direct relationship between turbidity and ciprofloxacin; and direct relationships between electrical conductivity, salinity, total dissolved salts, and acetaminophen. The last two parameters showed direct relationships with metronidazole but an inverse relationship with ciprofloxacin. The risk quotient for the detected pharmaceuticals showed low toxicity exposure (< 0.10) to algae, daphnids, and fish, except ciprofloxacin, which showed high toxicity exposure (> 1.00) to algae. The physicochemical properties of hospital effluents affect the concentrations and environmental risks of their constituents' analgesics and antibiotics.

## 1. Introduction

Pharmaceutical usage differs among regions on the basis of their application. Commonly used classes of pharmaceuticals include analgesics and their adjuvants (methylxanthines), and antibiotics, as most hospitals prescribe and administer them to patients [1–3]. Methylxanthines such as caffeine are usually added to some analgesics for their stimulatory effect and therefore considered analgesic adjuvants. Analgesics together with their adjuvants include over-the-counter drugs used for relieving pain and inflammation [4, 5], though they may exhibit antipyretic, anticoagulatory, anti-inflammatory, antithrombotic, or narcotic effects when administered to humans [6, 7]. Their presence in the environment may cause behavioral changes, growth and developmental impediments, reproductive defects, and lethal and population reduction effects in lower organisms [8–11]. Antibiotics are medicines used for treating and preventing bacterial infections by hindering growth of bacteria or eliminating them [12]. Their occurrence in the environment leads to antibiotic resistance, delays germination, lowers biomass allocation, and alters the composition of plant species [13–17]. The absorption of antibiotics by plants affects photosynthesis and mitochondrial functioning [18].

The suitability of water for proper functioning of aquatic ecosystems is determined by its physicochemical properties, which are quantified to serve as indicators of water quality [19]. Prior to disposal in aquatic environments, effluents should meet certain water quality standards [20]. Apart from the pollutants commonly borne by effluents, hospital effluents contain pharmaceutically active compounds (PhACs) and their metabolites [21–24], rendering them as major sources of pharmaceutical contamination in environmental compartments [25, 26]. They contaminate the environment with pharmaceuticals when treated (through wastewater treatment plants) or untreated, as in the case of most developing countries [24, 27]. Due to their ability to withstand degradation, active pharmaceutical ingredients (APIs) can persist in the environment and bioaccumulate along food chains [10]. Their presence in the environment is of global concern requiring immediate attention because of the risk they pose [28–30]. Unlike other environmental contaminants (potentially toxic elements and pesticides), pharmaceuticals have not received much attention [31, 32]. In Africa, most studies regarding pharmaceuticals centre on their occurrence in water resources and wastewater in general and not hospital effluents [33, 34]. Consequently, inadequate information exists on the monitoring of pharmaceuticals as contaminants. While Küster and Adler [35] suggest environmental risk assessment (ERA) as a way of dealing with the menace, Gani et al. [36] report that it provides insight into the alleviation and prioritization of such emerging contaminants.

ERA of pharmaceuticals is a legal requirement concerned with the determination of their potential detrimental consequences on the environment with the aim of taking precautionary measures for their management [37–39]. It gives an indication of the exposure to and effect of pharmaceuticals as contaminants and aids in safeguarding environmental health [40, 41]. When such information exists, a 5-year periodic review is required to ascertain the current risk [42]. Pharmaceutical concentrations in effluents can be influenced by a number of factors including physicochemical parameters of the effluents [43, 44]; it therefore follows that the environmental risks they pose can be affected by such parameters. Generally, little information exists on the relationship between physicochemical parameters of effluents and their pharmaceutical concentrations, as confirmed by Ohoro et al. [43], and subsequently the relationship between these parameters and environmental risks of pharmaceuticals. This study therefore seeks to evaluate the relationship between physicochemical parameters and environmental risks posed by commonly administered analgesics and antibiotics in hospital effluents in order to inform policies on environmental protection regarding pharmaceuticals as environmental contaminants.

## 2. Materials and Methods

### 2.1. Chemicals

Seven pharmaceuticals (four analgesics and three antibiotics) listed on the Ghana Essential Medicines List and the 2021 National Health Insurance Drug List [45, 46] were studied. They are acetaminophen (CAS #: 103-90-2), caffeine (CAS #: 58-08-2), diclofenac (CAS #: 15307-86-5), ibuprofen (CAS #: 15687-27-1), amoxicillin (CAS #: 26787-78-0), ciprofloxacin (CAS #: 85721-33-1), and metronidazole (CAS #: 443-48-1) purchased from Kinapharma Limited, Ghana, in their pure forms. The frequency of application in Ghana and inclusion in previous studies informed the choice of pharmaceuticals under study [47–50]. The chemical structures and physicochemical properties of the selected pharmaceuticals are shown in Table 1. Reagents used were trifluoroacetic acid (TFA), acetonitrile, methanol (high-performance liquid chromatography [HPLC] grade), and standard solutions of the pharmaceuticals, obtained by dissolution of pure samples in methanol and kept in a freezer at −18°C.

### 2.2. Study Sites

**Figure 1:**
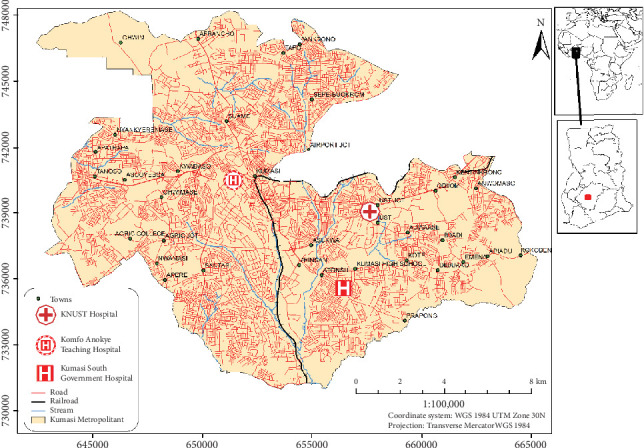
Map of Kumasi showing the three selected hospitals.

The study was carried out in three selected hospitals within Kumasi, the capital city of Ashanti Region, Ghana (Figure 1), which is equipped with 136 health facilities that provide healthcare to its citizens [58]. The hospitals selected for the study were Kwame Nkrumah University of Science and Technology (KNUST) Hospital (district level), Kumasi South Hospital (KSH) (secondary level), and Komfo Anokye Teaching Hospital (KATH) (tertiary level). The levels show the degree of care provided and the scope of jurisdiction. KNUST Hospital is classified as a district hospital that provides the healthcare needs of the university community and catchment area [59, 60]. KSH, located at Chirapatre Agogo, is a regional hospital and the second largest in the southern part of the Ashanti Region which provides the healthcare needs of various communities surrounding Atonsu while serving as a clinical centre for learning and research [61, 62]. KATH in the Ashanti Region is a tertiary teaching hospital that accepts referrals from 12 administrative regions in Ghana [63, 64].

### 2.3. Sample Collection

Gloves, nose mask, and sneakers were worn to prevent contamination and ensure safety. Samples were collected directly into precleaned and effluent-rinsed 1500 mL polyethylene bottles where possible, or fetched with a precleaned and effluent-rinsed cup and transferred into 1500 mL polyethylene bottles, or transferred into precleaned and effluent-rinsed plastic bucket and thoroughly mixed (for composite sampling) at available and accessible collection points before transferring into precleaned and effluent-rinsed 1500 mL polyethylene bottles. Sample collection was done on three different days of visit from the same sampling sites at each hospital. Three replicates of composite untreated effluent samples were obtained from each sampling point: septic tanks from children's male, female, and maternity wards; Osei Tutu Medical Complex (OTMC); and the laboratory and theatre at KNUST Hospital. At KSH, three replicates of composite untreated effluent samples were obtained from two main wastewater pipes. Three replicates of terminal untreated effluent samples were obtained from the end point of KATH's sewerage system prior to conveyance out of its premises. Composite and terminal samplings were used depending on the disposal practice of the hospital and the accessibility of the sampling site. Without any order of importance or reference, samples were collected on the 12th, 25th, and 26th of April 2022 at KNUST Hospital; the 25th and 26th of April 2022 and 10th of May 2022 at KSH; and the 30th of May 2022 and 27th and 28th of June 2022 at KATH; were kept in an ice chest at a temperature below 4°C, and transported to the Central Laboratory of KNUST, where they were stored in a refrigerator. A total of 27 samples (9 from each hospital) were collected.

### 2.4. Sample Preparation

Effluent samples were filtered using a 125-mm Prat Dumas filter paper to remove particulate matter. Five hundred milliliters of portions of the filtered effluent samples was used for the determination of physicochemical parameters (temperature, pH, electrical conductivity [EC], salinity, turbidity, and total dissolved salts [TDSs]) and pharmaceuticals under study. For interference removal and analyte concentration, portions of the remaining filtered effluent samples were loaded onto a hydrophilic–lipophilic balance solid-phase extraction (SPE) cartridge (6 mL, 200 mg Green Mall, Jinangsu, China). Adjustments were made to the SPE protocol as employed by Azanu et al. [31, 65] and used for the study. Four milliliters of methanol was used to condition the cartridges followed by equilibration with 4 mL of distilled water. Four hundred milliliters of samples of the filtered effluent was loaded onto the SPE cartridges, which were then washed with 5 mL of distilled water and eluted with 2 mL HPLC grade methanol. The eluted specimens were dried overnight and reconstituted in 2 mL of 1% methanol diluent before being filtered through 0.45 m membrane filters into HPLC vials for analysis.

### 2.5. Analysis of Physicochemical Parameters

Physicochemical parameters of effluent samples were determined at the Environmental Science Department Laboratory, KNUST. One hundred milliliters of each 500 mL filtered sample (meant for physicochemical parameters) was poured into a 150-mL beaker, and its temperatures, ECs, salinities, and TDS were determined by immersing a multiparameter probe (OHAUS) in it for 20 s, with the individual parameter readings recorded for each parameter of interest. The multiparameter probe was calibrated using potassium chloride. The meter has different probes for measuring the various parameters; therefore, the mode for particular parameters was selected, and its corresponding readings were recorded. A pH meter (STARTER3100 OHAUS) was used to determine the pH values, and a turbidimeter (VELP Scientifica) was used to measure the turbidity readings of the various effluent samples.

### 2.6. HPLC Analysis

Modifications were made to the analytical method for the detection of pharmaceutical residues in environmental samples as applied by Gyesi et al. [66]. The modifications involved adjustments to parameters like pH, temperature, mobile phase composition, choice of stationary phase or column, elution gradient, and detection wavelengths to achieve optimum chromatographic conditions for the detection of the pharmaceuticals under study.

#### 2.6.1. Analytical Method

The aforementioned analgesics and antibiotics in effluent samples were determined in a multicomponent analysis on a Germini C18 (150 × 4.6 mm, 5 *μ*m) column by elution gradient using PerkinElmer Flexar HPLC equipment coupled with a binary pump, autosampler, online degasser, and a photodiode array detector with Chromera analytical software. These components and chromatographic conditions adjusted in a systematic manner for optimum results as suggested by Choudhary [67] and Sabir et al. [68]. The mobile phase is comprised of A (0.05% TFA) and B (acetonitrile). The gradient composition was linearly changed from 90% A and 10% B to 65% A and 35% B in 5 min. After 4 min, a further linear alteration of 25% A and 75% B was made. This gradient composition was maintained for 7 min before a change to the initial ratio of 90% A and 10% B in 0.1 min and again maintained for the remaining 4 min. The injection volume was 20 *μ*L, and data was acquired at a wavelength of 320 nm for metronidazole, 275 nm for ciprofloxacin, and 225 nm for caffeine, diclofenac, ibuprofen, paracetamol, and amoxicillin. The flowrate was 1 mL/min, and the analysis was carried out at 30°C and a pH of 2.5.

#### 2.6.2. Validation of Analytical Method

The analytical method was validated against the ICH Guidelines [69] for linearity, limit of detection (LOD), limit of quantitation (LOQ), precision, accuracy, and robustness as specified by Choudhary [70] and Naseef et al. [71] to ensure quality. Seven solutions of 0.1% *w*/*v* of the pure pharmaceuticals under study were prepared as reference stock. Using HPLC grade methanol, six serial solutions at concentrations of 0.5, 1, 2, 4, 8, and 16 mg/L of each analyte were prepared to obtain 10 mL of solutions for each of the reference stock solutions. Twenty microliters of each solution was filtered with a membrane filter into 2 mL vials and analyzed with the HPLC equipment using the optimum chromatographic conditions developed. A 5-point calibration curve was drawn for each analyte across a 1000.00–32000.00 *μ*g/L concentration range to check the linearity of the developed method. The correlation coefficient (*r*^2^) for the calibration curves ranged from 0.9968 to 0.9997. The LOQ and LOD were determined from the standard deviation (*σ*) of the response from 1.00 *μ*g/mL antibiotics standard solution. The standard solution was injected six times, and the slope (S) of the calibration curve was determined. The LOQ and LOD were calculated from 10*σ*/S and 3.3*σ*/S, respectively [69]. Respective LODs and LOQs for the analytes were 890.00 and 2700.00 *μ*g/L for acetaminophen, 1150.00 and 3490.00 *μ*g/L for caffeine, 990.00 and 1830.00 *μ*g/L for diclofenac, 2400.00 and 7270.00 *μ*g/L for ibuprofen, 6840.00 and 20,700.00 *μ*g/L for amoxicillin, 2380.00 and 7210.00 *μ*g/L for ciprofloxacin, and 1240.00 and 3760.00 *μ*g/L for metronidazole.

The serial solutions obtained were analyzed under the specified chromatographic conditions at two different times (6-h interval) during the day (intraday) and on two different days (interday) for precision. Robustness was determined by altering flow rate, temperature, and wavelength. The relative standard deviations, determined using the analysis of the mean peak areas derived from the chromatograms for precision and robustness, were all less than 2%. Blank samples were spiked at concentrations (determined on the calibration curves of each analyte) of 6860.00, 7620.00, and 8380.00 *μ*g/L for acetaminophen; 3970.00, 4290.00, and 5100.00 *μ*g/L for caffeine; 2490.00, 2880.00, 3800.00 *μ*g/L of diclofenac; 3420.00, 5460.00, and 6050.00 *μ*g/L of ibuprofen; 6680.00, 8200.00, and 9200.00 *μ*g/L of amoxicillin; 4190.00, 4850.00, and 5940.00 *μ*g/L of ciprofloxacin; and 3700.00, 4170.00, and 4620.00 *μ*g/L of metronidazole. These solutions were run through the SPE protocol and analyzed using the developed HPLC method. The respective recovered concentrations were 5720.00, 6290.00, and 6850.00 *μ*g/L for acetaminophen; 3760.00, 4150.00, and 4340.00 *μ*g/L for caffeine; 1760.00, 2480.00, and 3300.00 *μ*g/L for diclofenac; 3010.00, 4580.00, and 5680.00 *μ*g/L for ibuprofen; 4980.00, 6610.00, and 7160.00 *μ*g/L for amoxicillin; 3030.00, 3760.00, and 3920.00 *μ*g/L for ciprofloxacin; and 3990.00, 3630.00, and 4450.00 *μ*g/L for metronidazole. The mean recoveries varied from 72.03% to 98.54% showing that the developed method is within acceptable standards [31].

### 2.7. Statistical Analysis

Data was organized as averages, relative standard deviations, percentages, and correlation coefficients, using Microsoft Office Excel 2013. This was to determine the linearity, precision, accuracy, robustness, LOD, and LOQ. One-way ANOVA was performed using Statistix 8 to depict the differences in the physicochemical parameters of the effluent samples from the selected hospitals. The Pearson correlation at 5% significance was calculated to establish relationships among physicochemical parameters and concentration of pharmaceuticals.

### 2.8. Risk Assessment

Potential adverse effects of the analgesics and antibiotics under investigation on the aquatic environment were assessed by an estimation of risk quotient (RQ) which is a ratio of maximum measured environmental concentration (MEC) to predicted no effect concentration (PNEC) as indicated by Belden [72] and Zhou et al. [73]. The PNEC values used for risk assessment in this study are consistent with the lowest ecotoxicological PNEC results calculated by the ecological structure-activity relationship (ECOSAR) model of US-EPA [74] as used by Azanu et al. [31]. As suggested by the European Chemicals Agency [75], RQs for three (3) trophic levels of the aquatic ecosystem, namely, algae, daphnids, and fish, were calculated in order to provide for a greater proportion of the aquatic food chain. The ranking criterion used for risk was as proposed by Zhou et al. [73] as environmental risk is low when RQ < 0.1, moderate when 0.1 < RQ < 1, and high when 1 < RQ < 10.

Antibiotic resistance risk was estimated as a ratio of measured inhibitory concentration (MIC) to assessment factor (AF). The lowest MIC (1%) values of detected antibiotics were obtained from the European Committee on Antimicrobial Susceptibility Testing (EUCAST) MIC database and an AF value of 10 as employed by Bengtsson-Palme and Larsson [76].

## 3. Results and Discussion

### 3.1. Physicochemical Characterization

**Figure 2:**
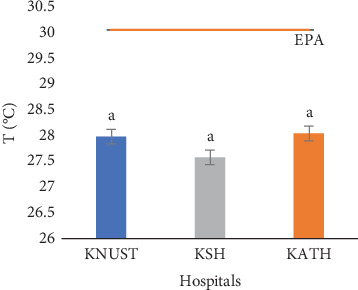
Temperature of effluents. T/°C, temperature in degrees Celsius; EPA, Environmental Protection Agency of Ghana.

**Figure 3:**
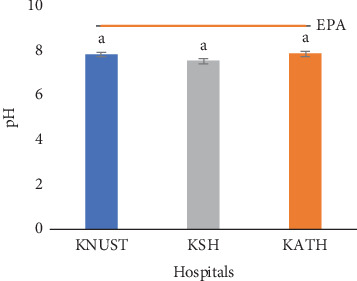
pH of effluents. EPA, Environmental Protection Agency of Ghana.

**Figure 4:**
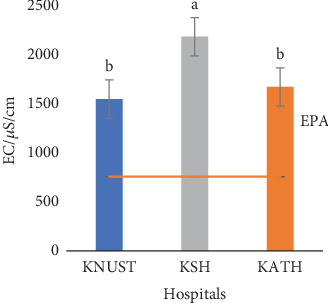
EC of effluents. EC, electrical conductivity; EPA, Environmental Protection Agency of Ghana.

The temperature readings for effluent samples from all the hospitals were close to 28.00°C with nonstatistically variations (*p* > 0.05) among samples from the three hospitals (Figure 2). It is likely the temperature of effluent samples is dictated by the ambient temperature, though it can be affected by the depth of sample collection [77]. The temperature of effluents is known to affect the physical, chemical, and biological processes of their recipient waterbodies [78]. The slightly above neutral pH values of the three hospitals' effluent samples as shown in Figure 3 might be due to the pH value of water as the concentrations of the pharmaceuticals detected were minute. Neutral pH values are ideal for plant growth, but the pharmaceuticals can accumulate along the food chain when absorbed by plants [79, 80]. High and low pH values, on the other hand, may corrode sewers and treatment plants [81]. The number and different concentrations of the pharmaceuticals detected in the three hospital effluents might account for their statistical differences (*p* ≤ 0.05) (Figure 3). Generally, the EC values for all the effluents were high showing the presence of dissolved salts. High EC can affect the survival of aquatic organisms and corrode sewer pipelines. The EC reading for KSH showed the highest value of 2163.30 *μ*S/cm, followed by KATH with a value of 1655.60 *μ*S/cm and then KNUST Hospital with a value of 1533.30 *μ*S/cm (Figure 4) Values for KNUST Hospital and KATH were not statistically different from one another but different from KSH samples (*p* < 0.05). These differences might be due to geochemical processes [82].

**Figure 5:**
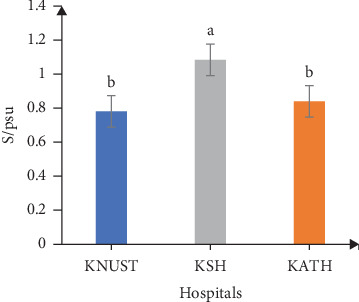
Salinity of effluents. S, salinity. EPA has no limit for salinity.

**Figure 6:**
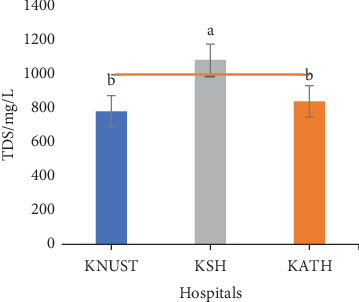
TDS of effluents. TDS, total dissolved salts; EPA, Environmental Protection Agency of Ghana.

**Figure 7:**
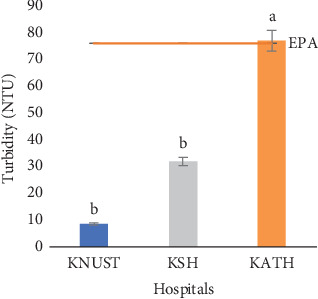
Turbidity of effluents. EPA, Environmental Protection Agency of Ghana.

Respective salinity readings for effluent samples were 0.78, 1.08, and 0.84 psu for KNUST Hospital, KSH, and KATH and were significantly different (*p* < 0.05) (Figure 5). The salinity values for KNUST Hospital and KATH were not statistically different from one another but however statistically different from KSH. The high salinity of KSH effluent samples implies the presence of a high content of dissolved salts, and this might be caused by the flow of such effluents through open drains leading to the possibility of dissolution of substances. The dissolved salts give rise to ions which have effect on the growth and reproduction of organisms [83]. TDS values recorded for effluent samples were in ascending order of 778.10, 836.10, and 1076.20 mg/L for KNUST Hospital, KATH, and KSH, respectively (Figure 6). Readings from KNUST Hospital and KATH were not statistically different from one another but significantly different from KSH readings (*p* < 0.05). High TDS affects the osmoregulation of aquatic organisms and can be detrimental [84]. The high EC and TDS values for KSH effluent samples may be linked to their high salinity values since according to Benit and Roslin [85], EC and TDS are indicators of salinity. Respective turbidity values of effluent samples from KNUST Hospital and KSH were 8.40 and 31.60 NTU (Figure 7). These values showed significant differences with KATH values being significantly different from KNUST Hospital and KSH values, though values for these latter ones are not significantly different. Turbidity is caused by suspended inorganic and organic suspended particles, and it correlates with microbial load [86]. Thus, the high turbidity of KATH could be attributed to the high production of effluents since it is a tertiary hospital, though erosion and surface runoffs as a result of rainfall could be contributing factors. High turbidity increases infection in the aquatic environment while the inverse decreases primary production thereby affecting chlorination during water treatment [87–89].

The temperature of the effluents was below the EPA limit of 30.00°C (Figure 2), while their pH values were within the EPA range of 6.00–9.00 (Figure 3). Figure 4 shows that ECs of the effluent samples exceeded the EPA limit of 750.00 *μ*S/cm for discharged wastewater, an indication of high inorganic substance dissolution, hence the possibility of the effluent to pollute the recipient aquatic environment [90]. There is no EPA limit for salinity in Ghana, hence no indication in Figure 5. It can be seen from Figure 6 that the EPA limit value (1000.00 mg/L) for TDS is higher than the values of KNUST Hospital and KATH but slightly lower than that for KSH. Figure 7 shows that turbidity readings for KNUST Hospital and KSH were lower than the EPA limit of 76.20 NTU while that of KATH is slightly higher than the EPA limit. The water quality of the hospital effluents does not fully meet the EPA standards.

### 3.2. Occurrence of Pharmaceuticals in Hospital Effluents

**Figure 8:**
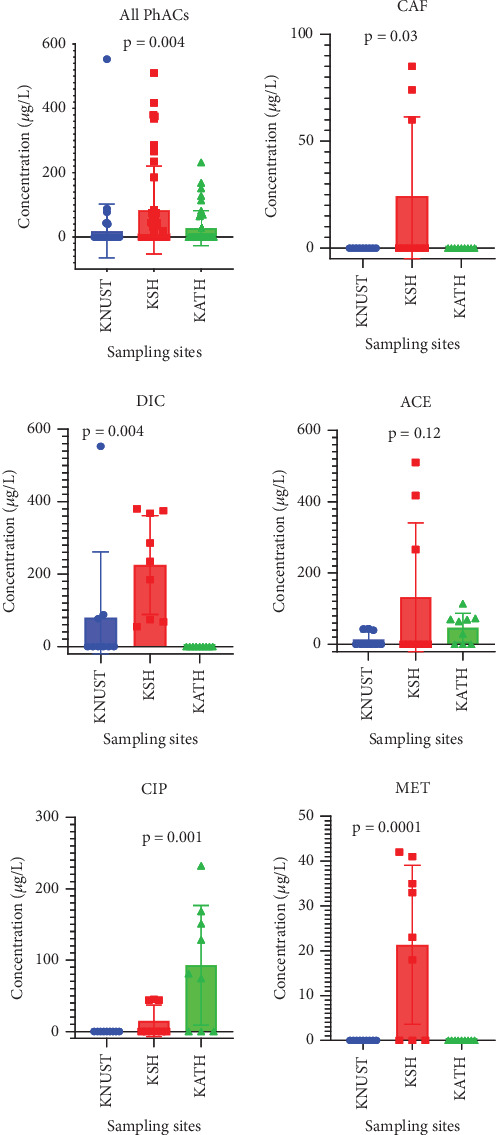
Concentration of pharmaceuticals in hospital effluents from sampling sites.

Acetaminophen and diclofenac were the analgesics detected in KNUST Hospital effluent samples with concentrations of 40.00–44.00 and 77.00–553.00 *μ*g/L, respectively, with the rest and antibiotics below detection levels (Figure 8). For KSH effluent samples, concentrations of analgesics detected were acetaminophen (266.00–510.00 *μ*g/L), caffeine (60.0–85.00 *μ*g/L), and diclofenac (55.00–380.00 *μ*g/L) while those of antibiotics were ciprofloxacin (44.00–45.00 mg/L) and metronidazole (18.00–42.00 *μ*g/L) (Figure 8). As can be seen in Figure 8, ibuprofen and amoxicillin were below the detection level. Apart from acetaminophen and ciprofloxacin detected at concentrations of 29.00–114.00 and 74.00–232.00 *μ*g/L, respectively, in effluent samples from KATH, the rest were all below detection levels (Figure 8). Similar to observations made by Azanu et al. [31] and Gyesi et al. [66], the concentrations of pharmaceuticals detected were generally low. Ibuprofen and amoxicillin were below detection level possibly because they are not administered due to respective development of tolerance and resistance for them. In addition, ibuprofen mostly occurs in sediments and soils while caffeine and diclofenac have often been found to occur in raw wastewater [91]. The differences in concentrations of pharmaceuticals detected might be due to the differences in the management of wastewater by the hospitals. Wastewater generated at KNUST Hospital is channeled through septic tanks via the waste treatment plant (KNUST wastewater treatment plant); that of KSH is discharged through open drains and septic tanks to an uninhabited waterlogged site (wetland) where there could be a possible purification of the effluents, while KATH employs a sewerage system connected to waste stabilization pond. Since the treated effluents from these three hospitals end in rivers, namely, Wiwi River for KNUST and KSH and Subin River for KATH, there could be routine assessments to check the quality of effluents discharged to safeguard the rivers.

Their occurrence might be through excretion as a result of incomplete or no metabolism after consumption or direct disposal. Their detection confirms reports that these classes of pharmaceuticals are rampant in hospital effluents and subsequently contaminate the environment [29, 49, 50, 92]. Thus, pharmaceuticals occur in hospital effluents rendering them as one of the origins of their contamination in the environment. These pharmaceuticals might be removed by biological and geological processes while others form metabolites as reported by Kosma et al. [50], Paíga et al. [93], and Reis et al. [25]. Also, pharmaceuticals and their metabolites might be persistent due to their physicochemical properties that render them stable or pseudopersistent as a result of continuous use and release into the environment [94]. Their presence in the environment might have adverse effects on nontarget aquatic organisms through bioaccumulation and subsequent interference with the normal functioning of biological systems. They can possibly be transported to wastewater treatment plants, surface waters, drinking water, and groundwater or get adsorbed onto soils.

**Figure 9:**
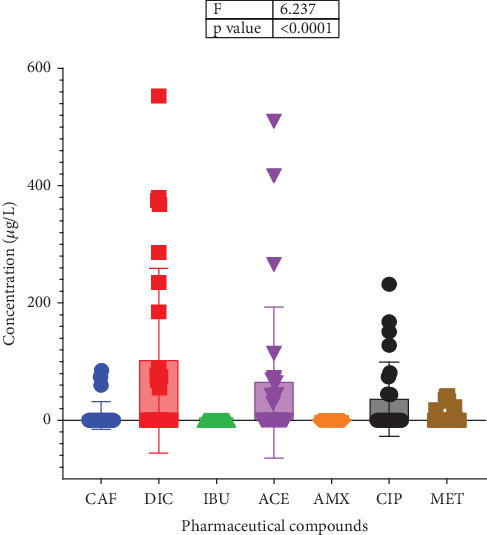
Concentration of pharmaceuticals in hospital effluents.

The order of detection of the analgesics was diclofenac > acetaminophen > caffeine > ibuprofen. Diclofenac is the analgesic with the highest detected concentration of 553.00 *μ*g/L in the KNUST Hospital effluent sample, followed by acetaminophen with a detected concentration of 510.00 *μ*g/L from the KSH effluent sample and then caffeine with a detected concentration of 85.00 *μ*g/L from KSH effluent sample (Figures 8 and 9). These concentrations are significantly different from one another (Figures 8 and 9). The detection of diclofenac with the highest concentration is similar to findings by Gyesi et al. [66]. This is because it is the most commonly dispensed and used pain relief drug due to its effectiveness, hence its occurrence in two of the selected hospitals (KNUST Hospital and KSH). Acetaminophen, which follows as the second, is prevalent in all the selected hospitals since it is considered as a first line of medication, frequently used, and safe for pain relief. Caffeine was the least detected analgesic found in only one of the selected hospitals, possibly because it is a constituent of certain beverages and drinks and used in drugs as an adjuvant for its effectiveness [95, 96]. Ibuprofen concentration was below detection level (Figure 9) because it is less prescribed or used as compared to the rest of the analgesics.

The order of detection of antibiotics was ciprofloxacin > metronidazole > amoxicillin. Ciprofloxacin was detected with the highest concentration of 232.00 *μ*g/L in KATH effluent samples (Figures 8 and 9) due to its broad-spectrum nature and use for the treatment of multidrug-resistant bacterial infections. Metronidazole has a high tendency to be adsorbed onto sediment or soil particles [97], accounting for its least detection concentration of 42.00 *μ*g/L in KSH effluent sample (Figures 8 and 9). Amoxicillin's concentration was below the detection level because it is a first-line antibiotic and might have lost its effectiveness against most bacterial infections and therefore least used or prescribed. Thus, some pharmaceuticals get adsorbed to solids suspended in wastewater while the others (soluble ones) remain in solution. The presence of antibiotics in hospital effluents makes them more likely to be found in other environmental compartments which can serve as conducive growth media for antibiotic-resistant bacteria and genes. The presence of these pharmaceuticals seems to be widespread as Azanu et al. [31] and Serna-Galvis et al. [98] have reported similar findings in their respective localities.

### 3.3. Correlation Between Physicochemical Parameters and Pharmaceuticals

Correlations between the physicochemical parameters and pharmaceutical concentrations give an indication that the latter is impacted by the former. At a 0.05 level of significance, there was a moderate negative relationship of −0.66 between temperature and acetaminophen and a strong positive relationship of 0.94 between pH and acetaminophen as shown in Table 2. The inverse relationship between temperature and acetaminophen might be attributed to the biodegradability of pharmaceuticals at high temperatures as reported by Rozman et al. [99]. While high temperatures cause the persistence of pharmaceuticals in aquatic ecosystems, low temperatures increase their concentration in surface waters, depicting the link to seasonal variations [43]. Polarity or dissociation constant (pKa) or sorption of pharmaceuticals could lead to the inverse relationship depicted by pH and acetaminophen. Also, neutral pH is known to promote acetaminophen adsorption [100, 101]. Table 2 shows that EC, salinity, and TDS had a strong positive correlation of 0.90 each with acetaminophen and 0.79 and 0.77 for the last two parameters each with metronidazole. While the last two parameters showed a respective strong negative correlation of −0.75 and −0.72 with ciprofloxacin, turbidity exhibited a strong positive relationship of 0.63 with the same pharmaceutical. High EC has been associated with high pharmaceutical concentrations [43]. Salinity affects the dynamics of pharmaceuticals in effluents [102] and is associated with EC and TDS; hence, these physicochemical parameters exhibit the same relationships. It is possible that acetaminophen and metronidazole are less adsorbed to sediment as compared to ciprofloxacin as its relationship with turbidity depicts its possible suspension in the effluent. This confirms the report by Yang et al. [103] that the adsorption of pharmaceuticals in suspended particulate matter is higher than in the sediment-water phases.

The unique physicochemical properties of the pharmaceuticals under study could contribute to the correlations exhibited. Acetaminophen (an analgesic) is a 4-aminophenol acetyl compound with a pH of 6, whose solubility in water increases with temperature and its stability is affected by humidity, light, and pH [51]. Sokół et al. [104] suggest that a photochemical process involving hydroxyl radicals might account for the breakdown of acetaminophen. Metronidazole, an antibiotic with a pH of 6.5, is a nitroimidazole compound with moderate water solubility and is stable in light and under acidic conditions [57]. These properties make it persistent in the environment. Ciprofloxacin (an antibiotic) is an acidic fluoroquinolone with poor water solubility and is less stable [54]; hence, it can stay in suspension.

### 3.4. Risk Assessment

The maximum MEC values were used to estimate the RQ in order to consider the worst occurrence scenario. In Table 3, the maximum MECs for the detected pharmaceuticals in KNUST Hospital untreated effluents are in ascending order of 5.53*e* + 02*  μ*g/L for diclofenac and 4.40*e* + 01*  μ*g/L for acetaminophen with a corresponding RQ at the trophic levels under consideration being < 0.1 which is low risk as indicated by Zhou et al. [73]. For KSH effluents, the maximum MEC values recorded were in descending order of 5.10*e* + 02*  μ*g/L for acetaminophen, 3.80*e* + 02*  μ*g/L for diclofenac, 8.50*e* + 01*  μ*g/L for caffeine, 4.50*e* + 01*  μ*g/L for ciprofloxacin, and 4.20*e* + 01*  μ*g/L for metronidazole as shown in Table 4. With the exception of ciprofloxacin which showed a high toxicity risk for exposure to algae with RQ of 7.38*e* + 00 and low toxicity for exposure to daphnids and fish with RQ of 4.55*e* − 03 and 1.80*e* − 05, respectively, all other pharmaceuticals showed low toxicity exposure to algae, daphnid, and fish with RQ of < 1 (Table 4). The maximum MEC values of acetaminophen (1.14*e* + 02*  μ*g/L) and ciprofloxacin (2.32*e* + 02*  μ*g/L) detected in KATH untreated effluents registered RQ values of < 0.1 at all trophic levels except ciprofloxacin showing RQ for algae of 3.80*e* + 01 (Table 5). Findings show that analgesics and antibiotics pose different levels of risk to algae, daphnia, and fishes. Chen et al. [105] reported low-risk toxicity of acetaminophen while Zhou et al. [73], on the contrary, found caffeine and ciprofloxacin exhibiting a moderate toxicity risk and diclofenac depicting a high exposure toxicity. However, Azanu et al. [31] reported high-risk exposure toxicity of ciprofloxacin. The high risk of ciprofloxacin to algae is an indication of its potential hazards in aquatic environments, hence the need for immediate attention. Though the risks of other pharmaceuticals considered were low, there is the possibility of their exhibition of medium to high risk due to increased or continuous use. This is because a study by Ashfaq et al. [106] has shown that some of these pharmaceuticals like diclofenac and paracetamol pose a high risk, while ciprofloxacin which exhibited a high risk has been reported by Zhou et al. [73] to exhibit moderate risk.

MIC value used was 2.00 *μ*g/L for ciprofloxacin and 16.00 *μ*g/L for metronidazole with their corresponding resistance risks as 116.00 and 2.63, respectively. Both ciprofloxacin and metronidazole exhibited resistance risk with the former being higher than the latter, a possible indication of their high use and/or abuse as suggested by the World Health Organization [107].

## 4. Conclusion

Hospital effluents show ambient temperature and around neutral pH but a high EC, salinity, TDSs, and turbidity and do not fully meet the required water quality standards for disposal. Additionally, they bear analgesics and antibiotics as contaminants and could be sources of pharmaceutical contamination in the environment, an indication of the demand for their unique treatment before disposal. The study showed an inverse relationship between temperature and acetaminophen and pH and acetaminophen but a direct relationship between EC and acetaminophen, salinity and acetaminophen, and TDS and acetaminophen. The relationships between salinity and metronidazole as well as TDS and metronidazole were direct. There was an inverse relationship between salinity and ciprofloxacin and TDS and ciprofloxacin but a direct relationship between turbidity and ciprofloxacin. These correlations prove that the physicochemical parameters of hospital effluents affect their constituents' analgesic and antibiotic concentrations. Aside water quality, other factors like drug properties, climate, naturally occurring environmental processes, and wastewater management practices affect the concentrations of analgesics and antibiotics in hospital effluents. By exhibiting some level of risk, both analgesics and antibiotics pose hazards to aquatic organisms with ciprofloxacin showing high risk to algae. Additionally, antibiotics exhibit resistance risk with ciprofloxacin having a higher resistance risk than metronidazole. Consequently, the occurrence of analgesics and antibiotics in hospital effluents poses environmental risks to aquatic life and subsequently human health. Such risk is influenced by the factors that affect the concentrations of analgesics and antibiotics in hospital effluents. Managing these factors with the aim of reducing or eliminating the occurrence can lead to the abatement of the environmental risk and its negative consequences.

## Figures and Tables

**Table 1:** Chemical structure and physicochemical properties of selected pharmaceuticals.

**Pharmaceuticals**		**ID**	**Structure**	**Molecular formula**	**Molecular weight**	**LogKow**	**pKa**	**Solubility (mg/L)**
Analgesics	Acetaminophen	ACE	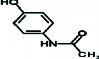	C_8_H_9_NO_2_	151.16	0.46	9.38	14,000.00
	Caffeine	CAF	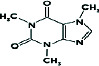	C_8_H_10_N_4_O_2_	194.19	−0.07	14.00	21,600.00
	Diclofenac	DIC	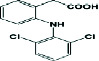	C_14_H_11_Cl_2_NO_2_	296.15	4.51	4.20	2.37
	Ibuprofen	IBU	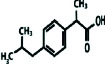	C_13_H_18_O_2_	206.28	3.97	4.91	21.00

Antibiotics	Amoxicillin	AMX	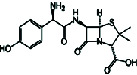	C_16_H_19_N_3_O_5_S	365.40	0.87	3.20 11.70	3430.00
	Ciprofloxacin	CIP	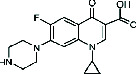	C_17_H_18_FN_3_O_3_	331.34	0.28	6.09 8.74	36,000.00
	Metronidazole	MET	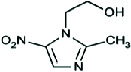	C_6_H_9_N_3_O_3_	171.15	−0.02	2.38	11,000.00

*Note: Source:* [51–57].

**Table 2:** Correlation between physicochemical parameters and pharmaceuticals.

	**Acetaminophen**	**Caffeine**	**Diclofenac**	**Ibuprofen**	**Amoxicillin**	**Ciprofloxacin**	**Metronidazole**	**C/*μ*S/cm**	**T/°C**	**S/psu**	**TDS/mg/L**	**Turbidity (NTU)**	**pH**
Acetaminophen		−0.56	0.99⁣^∗^	NA	NA								
Caffeine				NA	NA								
Diclofenac		−0.52		NA	NA								
Ibuprofen	NA	NA	NA	NA	NA	NA	NA	NA	NA	NA	NA	NA	NA
Amoxicillin	NA	NA	NA	NA	NA	NA	NA	NA	NA	NA	NA	NA	NA
Ciprofloxacin	0.45		0.74	NA	NA								
Metronidazole			−0.24	NA	NA	0.98							
C/*μ*S/cm	0.90⁣^∗∗∗^	−0.92	0.03	NA	NA	−0.75⁣^∗∗^	0.79⁣^∗^						
T/°C	−0.66⁣^∗^	−0.90	−0.39	NA	NA	0.18	0.64	−0.29					
S/psu	0.90⁣^∗∗∗^		−0.02	NA	NA	−0.72⁣^∗^	0.77⁣^∗^	0.99⁣^∗^	−0.3				
TDS/mg/L	0.90⁣^∗∗∗^	−0.90	0.03	NA	NA	−0.72⁣^∗^	0.77⁣^∗^	1.00⁣^∗^	−0.28	1.00			
Turbidity (NTU)	−0.33	0.07	−0.15	NA	NA	0.63⁣^∗^	−0.52	−0.2	0.18	−0.21	−0.21⁣^∗∗∗^		
pH	−0.94⁣^∗∗∗^	−0.71	0.33	NA	NA	0.56	−0.17	−0.32	0.19	−0.29	−0.29	0.02	

*Note:* C, electrical conductivity; NA, not applicable because concentrations were below detection levels; S, salinity; T, temperature in degrees Celsius.

Abbreviation: TDS, total dissolved salts.

⁣^∗^*p* < 0.05–0.001.

⁣^∗∗^*p* < 0.001–0.0001.

⁣^∗∗∗^*p* < 0.0001–infinity.

**Table 3:** Risk assessment of analgesics and antibiotics in KNUST Hospital effluents.

**Pharmaceuticals**		**MEC (*μ*g/L)**	**PNEC algae (*μ*g/L)**	**RQ algae**	**PNEC daphnids (*μ*g/L)**	**RQ daphnids**	**PNEC fishes (*μ*g/L)**	**RQ fishes**
Analgesics	Caffeine	NA	1.27*e* + 06	NA	3.46*e* + 03	NA	7.22*e* + 03	NA
	Diclofenac	5.53*e* + 02	4.14*e* + 04	1.34*e* − 02	2.58*e* + 01	2.15*e* − 02	3.77*e* + 01	1.47*e* − 02
	Acetaminophen	4.40*e* + 01	8.30*e* + 05	5.30*e* − 05	2.16*e* + 03	2.04*e* − 05	4.46*e* + 03	9.87*e* − 06

Antibiotics	Ciprofloxacin	NA	6.10*e* + 00	NA	9.90*e* + 00	NA	2.50*e* + 03	NA
	Metronidazole	NA	2.90*e* + 04	NA	3.20*e* + 01	NA	8.80*e* + 01	NA

*Note:* NA, not applicable because concentrations were below detection levels.

**Table 4:** Risk assessment of analgesics and antibiotics in KSH effluents.

**Pharmaceuticals**		**MEC (*μ*g/L)**	**PNEC algae (*μ*g/L)**	**RQ algae**	**PNEC daphnids (*μ*g/L)**	**RQ daphnids**	**PNEC fishes (*μ*g/L)**	**RQ fishes**
Analgesics	Caffeine	8.50*e* + 01	1.27*e* + 06	6.67*e* − 05	3.46*e* + 03	2.46*e* − 05	7.22*e* + 03	1.18*e* − 05
	Diclofenac	3.80*e* + 02	4.14*e* + 04	9.18*e* − 03	2.58*e* + 01	1.48*e* − 02	3.77*e* + 01	1.01*e* − 02
	Acetaminophen	5.10*e* + 02	8.30*e* + 05	6.15*e* − 04	2.16*e* + 03	2.36*e* − 04	4.46*e* + 03	1.14*e* − 04

Antibiotics	Ciprofloxacin	4.50*e* + 01	6.10*e* + 00	7.38*e* + 00	9.90*e* + 00	4.55*e* − 03	2.50*e* + 03	1.80*e* − 05
	Metronidazole	4.20*e* + 01	2.90*e* + 04	1.45*e* − 03	3.20*e* + 01	1.31*e* − 03	8.80*e* + 01	4.77*e* − 04

**Table 5:** Risk assessment of analgesics and antibiotics in KATH effluents.

**Pharmaceuticals**		**MEC (*μ*g/L)**	**PNEC algae (*μ*g/L)**	**RQ algae**	**PNEC daphnids (*μ*g/L)**	**RQ daphnids**	**PNEC fishes (*μ*g/L)**	**RQ fishes**
Analgesics	Caffeine	NA	1.27*e* + 06	NA	3.46*e* + 03	NA	7.22*e* + 03	NA
	Diclofenac	NA	4.14*e* + 04	NA	2.58*e* + 01	NA	3.77*e* + 01	NA
	Acetaminophen	1.14*e* + 02	8.30*e* + 05	1.37*e* − 04	2.16*e* + 03	5.28*e* − 05	4.46*e* + 03	2.56*e* − 05

Antibiotics	Ciprofloxacin	2.32*e* + 02	6.15*e* − 004	3.80*e* + 01	9.90*e* + 00	2.34*e* − 02	2.50*e* + 03	9.28*e* − 05
	Metronidazole	NA	2.90*e* + 04	NA	3.20*e* + 01	NA	8.80*e* + 01	NA

*Note:* NA, not applicable because concentrations were below detection levels.

## Data Availability

Data availability is not applicable in this study.
